# PPA Tele-Savvy: Developing an Online Intervention with Caregivers of Persons with Primary Progressive Aphasia

**DOI:** 10.1093/geroni/igab046.2965

**Published:** 2021-12-17

**Authors:** Darby Morhardt, Angela Roberts, Alyssa Penn, Allison Lindauer, Emily Rogalski, Sandra Weintraub, Kenneth Hepburn

**Affiliations:** 1 Northwestern University, Chicago, Illinois, United States; 2 Northwestern University, Evanston, Illinois, United States; 3 Oregon Health Sciences University, Portland, Oregon, United States; 4 Emory University, Atlanta, Georgia, United States

## Abstract

Primary progressive aphasia (PPA) typically results from a neurodegenerative disease such as frontotemporal lobar degeneration or Alzheimer’s disease and is characterized by a progressive loss of specific language functions with relative sparing of other cognitive domains until later stages when widespread cognitive decline consistent with generalized dementia is more prevalent. PPA tends to appear earlier than most dementias, in late middle-age, and can result in a high degree of psychological and economic burden for the family. Thematic analysis of PPA caregiver studies reveal families are learning to adapt to not only declining language across communicative contexts and domains, but concomitant behavioral, social communication and personality changes over time. While there are several evidence-based dementia caregiver interventions, none are specifically designed for the PPA family caregiver. This pilot project, funded by the Emory University Roybal Center is the adaptation of an evidence-based on-line psychoeducation program (Tele-Savvy) to address the unique challenges facing informal caregivers of those living with PPA and to help these caregivers achieve mastery within this context. PPA caregivers have been engaged through focus groups to identify their most pressing caregiving challenges and how the existing Tele-Savvy curriculum should be adapted to meet their needs. Synchronous and asynchronous video modules have been designed to address: PPA education, the impact on dyadic connection and caregiving challenges and communication strategies specific to PPA. The Tele-Savvy central processes of coaching and de-briefing will also be pilot tested and refined.

